# Early Summer Meningoencephalitis: Unusual yet Usual Diagnostic Challenge in a Geriatric Patient—A Case Report

**DOI:** 10.3390/reports9030205

**Published:** 2026-06-28

**Authors:** Georgiana Ciobanu, Daniel Pichler, Benjamin Hutter, Thomas Münzer

**Affiliations:** 1 Geriatric Clinic, Cantonal Hospital of St. Gallen, Health Ostschweiz Ostschweiz, 9007 St. Gallen, Switzerland; 2 Regional Hospital La Carità, 6600 Locarno, Switzerland; 3 Blood Donation Foundation Stiftung Blutspende Schweizerische Rote Kreuz Ostschweiz, 9000 St. Gallen, Switzerland; 4 Medbase Friedtalweg, 9500 Wil, Switzerland

**Keywords:** tick-borne encephalitis, geriatrics, multisystem disease, differential diagnosis

## Abstract

**Background and Clinical Significance:** When diverse clinical presentations coincide with complex laboratory findings, particularly in older adults, the diagnostic process can be especially challenging. **Case Presentation:** We report the case of a geriatric patient who was hospitalized with initial gastrointestinal and respiratory symptoms, followed by progressive chest pain and profound weakness, accompanied by elevated transaminases, troponin elevation, and hyponatremia, initially suggesting multiple competing diagnostic entities. During the clinical course, the patient developed neurological symptoms. Ultimately, careful history-taking, including detailed exposure assessment, raised suspicion for tick-borne encephalitis, which was subsequently confirmed by serological testing. **Conclusions:** This case highlights the diagnostic complexity of tick-borne encephalitis in older adults, where atypical and multisystem presentations may obscure the underlying etiology and delay recognition of a neuroinfectious disease.

## 1. Introduction and Clinical Significance

Tick-borne encephalitis (TBE) is a viral zoonotic infection caused by the tick-borne encephalitis virus (TBEV), a member of the Flavivirus genus, which is transmitted through the bite of infected Ixodes ticks. The disease is endemic in large parts of Europe and Asia, with marked geographical variation in incidence. In Switzerland, TBE risk areas encompass nearly the entire country, with the exception of the canton of Ticino. Germany is also considered endemic, particularly in the southern federal states of Bavaria and Baden-Württemberg, where the Black Forest region represents one of the most important TBE risk areas. The incidence is highest during the warm season, typically from April to November, reflecting tick activity patterns. Patients over 50 years of age are most frequently affected and often present with atypical symptoms and variable disease courses, which may contribute to diagnostic challenges [1,2,3].

Here, we present the case of a 71-year-old woman with a complex, multisystem prodrome encompassing gastrointestinal, respiratory, cardiac, and neurological symptoms, accompanied by overlapping, dynamic laboratory abnormalities.

Clinical Significance: This case highlights the diagnostic challenges of Tick-Borne Encephalitis (TBE) in geriatric patients, demonstrating how a complex, multisystem prodrome can mask the typical presentation. It underscores the critical necessity of meticulous exposure history, repeated clinical reassessments, and a high index of suspicion to avoid diagnostic delays when initial laboratory and clinical findings are misleading.

## 2. Case Presentation

A 71-year-old woman was referred to a tertiary care hospital with a three-day history of cough, interscapular pain, and intermittent diarrhea. Upon presentation, she was afebrile, and the physical examination revealed no significant abnormalities. Initial laboratory investigations were unremarkable, except for mildly elevated transaminases. Her medical history was notable for epilepsy of unclear etiology, arterial hypertension, and mild cognitive impairment. Home medications included aspirin, amlodipine, candesartan, carbamazepine, and lamotrigine, with no recent changes in dosage or treatment regimen. Her symptoms were initially interpreted as a nonspecific viral infection, and she was discharged.

Over the following days, the patient developed progressive weakness, nausea, vomiting, and upper abdominal pain, prompting readmission. Repeat laboratory testing demonstrated a further increase in transaminases (Table 1) without evidence of cholestasis, accompanied by a mild elevation in troponin levels.

Abdominal ultrasound and abdominal computed tomography imaging revealed no pathological findings. However, the patient was experiencing severe pain and shortness of breath. Given the differential diagnoses of pulmonary embolism and aortic dissection, a computed tomography angiography was performed, which showed no significant pathology; subsequent echocardiography also revealed no relevant abnormalities.

Given the clinical presentation and laboratory abnormalities, a broad differential diagnosis was explored during the diagnostic work-up (Table 2).

The patient remained stable and was transferred to our clinic with suspected acute gastroenteritis, associated hepatitis, and potential myocarditis. Shortly thereafter, she developed fever, symptomatic orthostatic hypotension with vertigo, and headache. Physical examination revealed new-onset diffuse sensory disturbances in the lower extremities and photophobia. Blood cultures remained negative, and SARS-CoV-2, influenza A/B, and urinary tract infections were excluded. Concurrently, euvolemic hypotonic hyponatremia (124 mmol/L), consistent with syndrome of inappropriate secretion of antidiuretic hormone, was detected and managed with fluid restriction.

During hospitalization, the patient reported a decline in memory and concentration. Her known mild cognitive impairment appeared to have slightly worsened, evidenced by a 3-point drop on the Montreal Cognitive Assessment (MoCA) scale. Upon repeated history-taking, she recalled intermittent headaches and vertigo starting one week prior to her initial presentation. Notably, she had gone hiking in the Black Forest (Germany) approximately two weeks before symptom onset. She did not recall a tick bite and was unvaccinated against tick-borne encephalitis (TBE).

The emergence of neurological symptoms prompted a reassessment of the differential diagnosis, with particular consideration of infectious causes of meningoencephalitis and other tick-borne diseases (Table 3).

The meningoencephalitic symptoms and the overall clinical course, combined with the absence of significantly elevated inflammatory markers, pointed toward viral meningitis as the most likely diagnosis. Based on the clinical presentation and exposure history, tick-borne encephalitis (TBE) was strongly suspected. The patient maintained a Glasgow Coma Scale (GCS) score of 15 without focal neurological deficits. Over the following days, she recovered spontaneously, with initial resolution of fever followed by improvement in sensory disturbances and fewer episodes of vertigo. In light of the clinical course, spontaneous clinical improvement, and the patient’s preferences, a lumbar puncture was not performed. Serological testing ultimately confirmed TBE infection, with positive IgM (>150 U/L) and IgG (>3000 U/L) titers.

Although the patient exhibited the typical biphasic pattern of TBE, her clinical course and laboratory evolution presented several unusual and noteworthy features. At follow-up, a gradual recovery of neurocognitive function back to her baseline level was observed.

**Figure 1:**
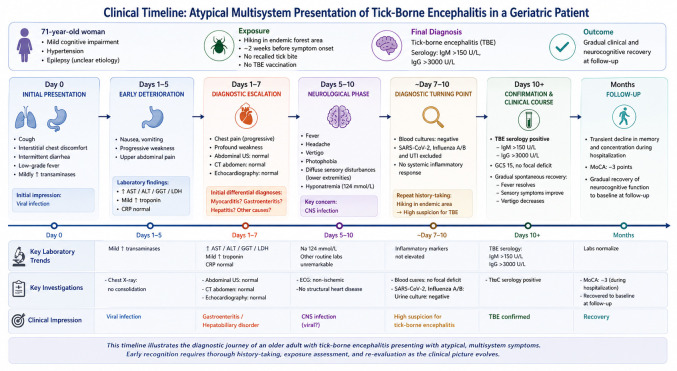
Clinical timeline and most prominent laboratory findings (↑: increased).

A timeline of the clinical course is shown in Figure 1.

## 3. Discussion

Clinically, tick-borne encephalitis (TBE) classically follows a biphasic course. It begins with a non-specific flu-like prodrome, followed—after a symptom-free interval—by neurological involvement ranging from meningitis to meningoencephalitis and, in severe cases, encephalomyelitis. However, this typical presentation occurs in only about 10% of cases globally, though it is reported more frequently in Europe (approximately 75%). While many cases, particularly in older adults, exhibit atypical, oligosymptomatic, or diagnostically misleading courses, potential complications include persistent balance disorders, cognitive impairment, paresis, and dysphagia. Rare instances of autonomic dysfunction (e.g., tachycardia, orthostatic hypotension) have also been described, as observed in our patient [4,5,6]. Furthermore, a positive correlation exists between increasing age and a poorer prognosis, with several studies investigating genetic predispositions to severe forms of meningoencephalitis [7,8].

In geriatric patients, the diagnostic challenge is further compounded by multimorbidity, pre-existing cognitive impairment, and the frequent coexistence of non-specific systemic symptoms and laboratory abnormalities. Initial findings such as transaminase elevation, hyponatremia, or troponin elevation may strongly mimic alternative hepatobiliary, cardiac, or gastrointestinal diseases, thereby delaying the consideration of neuroinfectious etiologies.

The importance of travel and exposure history is fundamental in the diagnostic evaluation of suspected tick-borne encephalitis (TBE), as it directly informs pre-test probability and guides clinical reasoning. The epidemiological context was particularly relevant in the present case. Although the patient reported a hiking trip in the Black Forest approximately two weeks before symptom onset, she is a resident of Switzerland, where TBE is endemic across most of the country. The Black Forest region is likewise recognized as one of the major TBE-endemic areas in Central Europe [3]. Given that the travel history represented a single identifiable exposure event, consideration of both the Swiss and German epidemiological settings was essential when assessing the likelihood of TBE.

Consequently, maintaining a broad differential diagnosis is essential, especially when diffuse clinical and laboratory findings initially suggest separate entities—such as hepatitis, myocarditis, or, more commonly, gastroenteritis. Conversely, once neurological symptoms raise suspicion of neuroinflammation, the full spectrum of infectious etiologies must be considered. This includes bacterial meningitis, enteroviruses, and herpes virus infections as critical diagnoses that must not be missed.

Diagnostic protocols for TBE are not yet fully standardized. In clinical practice, diagnosis relies primarily on serology. By the onset of neurological symptoms, viral RNA is typically no longer detectable in the blood or cerebrospinal fluid (CSF), rendering PCR useful only during the very early stages of infection. In contrast, specific IgM and IgG antibodies are reliably detectable in both serum and CSF during the meningoencephalitic phase [5]. Other laboratory anomalies are often non-specific, including moderately elevated transaminases (up to 80% of cases), hyponatremia (~30%), and elevated LDH (~12%), all of which typically normalize alongside clinical improvement [5,6].

Hyponatremia is a recognized laboratory abnormality in tick-borne encephalitis (TBE) and is most commonly attributed to the syndrome of inappropriate antidiuretic hormone secretion (SIADH), likely triggered by central nervous system inflammation and hypothalamic involvement. In some patients, hyponatremia may additionally reflect a broader stress-related neuroendocrine response during acute infection. The temporal association between the onset of neurological symptoms and the development of hyponatremia compatible with SIADH in our patient supports this pathophysiological link and suggests that the electrolyte disturbance was related to TBE. Although non-specific, hyponatremia may represent an additional supportive clue in the diagnostic work-up of TBE when interpreted within the appropriate epidemiological and clinical context.

Furthermore, mild cardiac involvement in tick-borne encephalitis (TBE) has been reported, although it is considered uncommon and is often underrecognized. Proposed mechanisms include direct viral involvement of the myocardium as well as indirect autonomic dysregulation during the acute neuroinfectious phase. These processes may lead to transient electrocardiographic abnormalities and, in rare cases, elevation of cardiac biomarkers such as troponin, even in the absence of clinically overt myocarditis. In our patient, the observed troponin elevation could therefore be interpreted in the context of systemic inflammatory response and possible neurocardiogenic involvement associated with TBE. Similar findings have been described in the literature, supporting the notion that TBE may occasionally be accompanied by reversible cardiac involvement [9].

Lumbar puncture is generally recommended in patients presenting with neurological symptoms, fever, and elevated inflammatory markers. The CSF profile in tick-borne encephalitis (TBE) typically reveals an initial granulocytic pleocytosis that subsequently shifts to a lymphocytic pleocytosis, accompanied by normal glucose levels and mildly elevated protein concentrations. CSF antibody testing, particularly when combined with calculation of the CSF/serum antibody index, can be especially valuable in previously vaccinated patients, as it may help confirm intrathecal antibody production and distinguish true infection from potential serological cross-reactivity with other flaviviruses [5].

In our case, an important limitation is that cerebrospinal fluid (CSF) examination was not performed. Consequently, the diagnosis in our patient relied primarily on serum serology, which carries inherent limitations, including the possibility of flavivirus cross-reactivity. However, interpretation of the serological findings was supported by the patient’s vaccination and exposure history, which were considered compatible with TBE infection.

The decision not to pursue lumbar puncture was made after careful consideration of the potential risks and benefits in this frail geriatric patient. The patient remained clinically stable throughout hospitalization, and her advanced age, known history of epilepsy, and expressed preference to avoid invasive diagnostic procedures were important factors in the shared decision-making process. In this context, the potential diagnostic yield of CSF examination was weighed against the procedural risks and the likelihood that the results would not substantially alter clinical management. This highlights the importance of individualizing diagnostic strategies in older adults, taking into account both clinical circumstances and patient preferences.

Another limitation is the concomitant use of antiepileptic medication, which may have influenced the neurological presentation. However, antiepileptic therapy remained unchanged throughout hospitalization, and renal function remained stable, making clinically relevant alterations in drug pharmacokinetics unlikely. Therefore, the observed neurological manifestations are unlikely to be attributable to changes in antiepileptic drug exposure.

Treatment remains purely supportive, as no specific antiviral therapy is currently available. Although corticosteroids are occasionally used to reduce cerebral edema in viral encephalitis, robust clinical evidence supporting their efficacy in TBE is still lacking [6,8].

Recent evidence suggests that viral neuroinflammation may accelerate cognitive decline in patients with pre-existing mild cognitive impairment (MCI) [10]. In our case, however, the patient exhibited only a transient worsening of neurocognitive function, with subsequent complete recovery to baseline. This transient cognitive decline may not be fully representative of the typical geriatric population, in whom TBE is more frequently associated with persistent and sometimes permanent neurological sequelae. At the same time, it is plausible that the observed impairment was multifactorial and related to the overall clinical context, including dizziness and systemic illness, rather than reflecting isolated direct viral neurocognitive injury. Indeed, the literature also describes a broad clinical spectrum of TBE, ranging from mild and self-limited forms to severe cases with long-term sequelae. Nevertheless, in older individuals affected by TBE, close monitoring of long-term neurological outcomes is warranted. Particular attention should be paid to the risk of persistent cognitive decline, secondary MCI, and other neurocognitive sequelae that can profoundly impair functional status and patient autonomy.

## 4. Conclusions

This case underscores how a multifaceted and atypical initial presentation can easily mask tick-borne encephalitis in older adults. It highlights that maintaining a high index of suspicion, performing meticulous exposure history-taking, and conducting continuous clinical reassessments are paramount to ensuring a timely and accurate diagnosis in geriatric patients.

## Figures and Tables

**Table 1:** Laboratory findings in the emergency department (first and second presentation).

Parameter	Reference	First Presentation	Second Presentation
Natrium (mmol/L)	136–144	137	133
Bilirubin total (umol/L)	<20	3	4
AST (U/L)	<55	84	330
ALT (U/L)	<55	69	201
GGT (U/L)	<35	444	754
ALP (U/L)	30–120	95	96
Total Proteine (g/L)	63–83	65	62
Albumin (g/L)	34–48	39.1	37.2
LDH (U/L)	<265	197	366
CK (U/L)	<145	41	75
Pancreatic Amylase (U/L)	<46	15	71
CRP (mg/L)	<8	2	<1
hs Troponin (ng/L)	<18	17.5	45.5

AST (Aspartate Aminotransferase), ALT (Alanine Aminotransferase), GGT (Gamma-Glutamyl Transferase), ALP (Alkaline Phosphatase), Total Protein, Albumin, LDH (Lactate Dehydrogenase), CK (Creatine Kinase), Pancreatic Amylase, CRP (C-Reactive Protein), and hs Troponin (high-sensitivity Troponin).

**Table 2:** Differential diagnoses considered during the initial diagnostic work-up of the patient, with findings supporting and arguing against each diagnosis in the present case.

Differential Diagnosis	Typical Symptoms	Findings Supporting the Diagnosis in This Case	Findings Arguing Against the Diagnosis in This Case
Acute viral gastroenteritis with reactive hepatitis	Diarrhea, nausea, vomiting, abdominal pain, malaise	Initial gastrointestinal symptoms; elevated transaminases	Biphasic course; subsequent fever, headache, photophobia, sensory disturbances, and dizziness not adequately explained
Acute viral hepatitis (HAV/HEV)	Nausea, vomiting, abdominal pain, fatigue, elevated liver enzymes, occasionally fever	Marked transaminase elevation; positive HAV IgG and HEV IgG serology	No evidence of acute infection (negative HAV IgM, HEV IgM, and HEV RNA); neurological symptoms unexplained
Myocarditis	Chest pain, dyspnea, fatigue, arrhythmias	Mild troponin elevation; viral prodrome	Echocardiography and cardiac work-up unremarkable; no evidence of myocardial dysfunction
Acute coronary syndrome (ACS)	Chest pain, dyspnea, autonomic symptoms, elevated troponin	Elevated hs-troponin; interscapular/epigastric pain	No ischemic ECG changes; cardiac imaging negative; symptoms atypical
Pulmonary embolism	Dyspnea, chest pain, tachycardia, syncope	Elevated troponin may occur; thoracic pain	Triple-rule-out CT negative
Aortic dissection	Sudden chest or interscapular pain, neurological symptoms, elevated cardiac biomarkers occasionally	Pain between the shoulder blades prompted evaluation	Triple-rule-out CT excluded dissection

**Table 3:** Differential diagnoses considered at the onset of neurological symptoms, with findings supporting and arguing against each diagnosis in the present case (final diagnosis in bold).

Differential Diagnosis	Typical Symptoms	Findings Supporting the Diagnosis in This Case	Findings Arguing Against the Diagnosis in This Case
Herpes simplex encephalitis	Fever, altered mental status, seizures, focal neurological deficits	Headache, photophobia, history of epilepsy	No altered consciousness, seizures, or rapidly progressive encephalopathy
Pneumococcal meningitis	Fever, headache, meningism, altered consciousness, photophobia, nausea/vomiting	Fever, headache, photophobia, neurological symptoms	Subacute biphasic course; absence of severe meningeal syndrome and impaired consciousness; negative blood cultures
Enteroviral meningoencephalitis	Fever, headache, photophobia, gastrointestinal symptoms, meningitis/encephalitis	Biphasic viral illness with neurological manifestations	Less consistent epidemiology; absence of leukopenia frequently reported in severe adult cases
Neuroborreliosis	Headache, radicular pain, cranial nerve palsies (especially facial nerve), sensory symptoms	Tick exposure in endemic region; neurological symptoms	No facial palsy, radiculitis, or typical Bannwarth syndrome
**Tick-borne encephalitis (TBE)**	**Biphasic illness with initial flu-like and gastrointestinal symptoms followed by fever, headache, dizziness, photophobia, and neurological manifestations**	**Recent hiking in endemic area, biphasic disease course, hyponatremia, elevated transaminases, neurological symptoms, positive TBEV IgM and IgG serology**	**Absence of CSF confirmation (diagnostic limitation)**

## Data Availability

The data presented in this study are available on request from the corresponding author due to privacy and ethical restrictions.

## Abbreviations

The following abbreviations are used in this manuscript:

TBE	Tick-borne Encephalitis
CT	Computed Tomography
MCI	Mild Cognitive Impairment
MoCA	Montreal Cognitive Assessment
AST	Aspartate Aminotransferase
ALT	Alanine Aminotransferase
GGT	Gamma-Glutamyl Transferase
ALP	Alkaline Phosphatase
LDH	Lactate Dehydrogenase
CK	Creatine Kinase
CRP	C-Reactive Protein
hs Troponin	high-sensitivity Troponin
